# Metabolomics Provides New Insights into the Mechanisms of *Wolbachia*-Induced Plant Defense in Cotton Mites

**DOI:** 10.3390/microorganisms13030608

**Published:** 2025-03-06

**Authors:** Xinlei Wang, Sha Wang, Ali Basit, Qianchen Wei, Kedi Zhao, Feng Liu, Yiying Zhao

**Affiliations:** College of Agriculture, Shihezi University, Shihezi 832003, China; 20222012077@stu.shzu.edu.cn (X.W.); 20222012067@stu.shzu.edu.cn (S.W.); basitali27297@stu.shzu.edu.cn (A.B.); 13002637216@163.com (Q.W.); zhaokedibs@163.com (K.Z.)

**Keywords:** *Tetranychus turkestani*, *Wolbachia*, mite–cotton interaction, metabolomics

## Abstract

Endosymbiotic bacteria play a significant role in the co-evolution of insects and plants. However, whether they induce or inhibit host plant defense responses remains unclear. In this study, non-targeted metabolomic sequencing was performed on cotton leaves fed with *Wolbachia*-infected and uninfected spider mites using parthenogenetic backcrossing and antibiotic treatment methods. A total of 55 differential metabolites were identified, which involved lipids, phenylpropanoids, and polyketides. KEGG pathway enrichment analysis revealed seven significantly enriched metabolic pathways. Among them, flavonoid and flavonol biosynthesis, glycerophospholipid metabolism, and ether lipid metabolism showed extremely significant differences. In *Wolbachia*-infected cotton leaves, the flavonoid biosynthesis pathway was significantly up-regulated, including quercetin and myricetin, suggesting that the plant produces more secondary metabolites to enhance its defense capability. Glycerophosphocholine (GPC) and sn-glycerol-3-phosphoethanolamine (PE) were significantly down-regulated, suggesting that *Wolbachia* may impair the integrity and function of plant cell membranes. The downregulation of lysine and the upregulation of L-malic acid indicated that *Wolbachia* infection may shorten the lifespan of spider mites. At various developmental stages of the spider mites, *Wolbachia* infection increased the expression of detoxification metabolism-related genes, including gene families such as cytochrome P450, glutathione S-transferase, carboxylesterase, and ABC transporters, thereby enhancing the detoxification capability of the host spider mites. This study provides a theoretical basis for further elucidating the mechanisms by which endosymbiotic bacteria induce plant defense responses and expands the theoretical framework of insect–plant co-evolution.

## 1. Introduction

In long-term co-evolution, insects and their endosymbiotic bacteria have established close mutualistic relationships [1]. Endosymbiotic bacteria not only regulate the host insect’s nutritional and reproductive metabolism but also assist in resisting biotic and abiotic stresses, enhancing resistance to chemical pesticides, and improving adaptability to host plants [2,3]. The association between phytophagous insects and their endosymbiotic bacteria affects various interactions between insects and plants, such as expanding the range of host plants [4] or promoting host plant utilization by altering the plant’s chemical composition [5]. However, little is known about the role of endosymbiotic bacteria in manipulating plant defense responses. In the long-term co-evolutionary processes between insects and plants, plants have continuously evolved various defense mechanisms to resist insect feeding, while insects have developed counter-defense strategies to overcome plant defenses. In the arms race of plant defense and insect counter-defense during co-evolution, the exact role of endosymbiotic bacteria remained unclear. Different studies have different conclusions. Some studies suggested that endosymbiotic bacteria induced and activated plant defense responses, while others found that they inhibited plant defense responses, and some indicated that they have no significant effect on plant defense responses. At the same time, the specific mechanism by which endosymbiotic bacteria mediate host plant defense responses is also unclear, which greatly restricts our in-depth understanding of the interaction between insects and plants.

Frago et al. (2012) proposed that endosymbiotic bacteria might act as hidden “executors” influencing host interactions [6]. Subsequent studies have further explored their role in modulating plant defenses. Giron et al. (2014) and Groen et al. (2016) demonstrated that insect endosymbionts can suppress plant defense responses both directly and indirectly [7,8]. Similarly, Chung et al. (2013) found that symbiotic bacteria present in the oral secretions of the Colorado potato beetle inhibited plant defenses, thereby promoting beetle growth [9]. Su et al. (2015) also reported that *Hamiltonella defensa* infection in *Bemisia tabaci* suppressed jasmonic acid (JA)-mediated defenses in tomatoes, enhancing whitefly development [10]. In contrast, Staudacher et al. (2017) showed that although combinations of *Wolbachia*, *Cardinium*, and *Spiroplasma* affected plant defense parameters in *Tetranychus urticae*, no causal relationship was established between endosymbionts and induced plant defenses [11].

Recent research has delved into the molecular mechanisms underlying the endosymbiont-mediated modulation of plant defenses. Li et al. (2019) found that *H. defensa* infection in wheat suppressed key genes involved in SA and JA signaling pathways, reduced defense enzyme activities (PPO and POD), and enhanced detoxification enzyme activity in *Sitobion avenae* [12]. Zhu et al. (2020) reported that *Wolbachia* and *Spiroplasma* infections in *T. truncatus* altered tomato defense enzyme activities and gene expression but did not significantly impact JA and SA accumulation, suggesting that regulation may occur downstream of JA and SA biosynthesis [13]. Most of these studies focused on defense gene expression, enzyme activities, or hormone accumulation.

However, single-molecule approaches often fall short of explaining complex biological responses, which typically involve intricate interactions among genes, proteins, and metabolites. Omics technologies, such as metabolomics, provide a comprehensive approach to identify biomolecules in complex samples. Since changes in small molecular metabolites can directly reflect the physiological state of plants, particularly in plant secondary metabolites, metabolomics offers a powerful tool to explore these complex interactions. Therefore, this study aims to investigate the role of endosymbionts in mediating plant defenses using metabolomics, thereby providing new insights into the molecular mechanisms underlying endosymbiont-induced plant responses, especially concerning secondary metabolites.

Xinjiang is the largest cotton production region in the world. *Tetranychus turkestani*, belonging to the order Trombidiformes, family Tetranychidae, and genus Tetranychus, is the dominant pest species in Xinjiang cotton fields [14]. *Wolbachia* is a widespread symbiotic bacteria found in arthropods, classified under the class α-Proteobacteria, order Rickettsiales, family Anaplasmataceae, and genus *Wolbachia* [15]. It can infect up to 70% of arthropod species [16], causing various reproductive regulations in hosts, such as cytoplasmic incompatibility [17]. We hypothesize that *Wolbachia* infection in *T. turkestani* alters the metabolic profile of cotton leaves, particularly affecting secondary metabolites involved in plant defense mechanisms. To test this hypothesis, *T. turkestani* strains infected and uninfected with *Wolbachia* were obtained through parthenogenetic backcrossing and antibiotic treatment. These strains were used to infest cotton leaves, followed by metabolomics analysis to identify significantly differential metabolites. The functions and pathways of key differential metabolites, particularly secondary metabolites linked to plant chemical defense, were further validated. This research aims to systematically elucidate the molecular mechanisms of endosymbiont-induced plant defense from a metabolic perspective, thereby contributing to the advancement of theories on insect–plant co-evolution.

## 2. Materials and Methods

### 2.1. Experimental Materials

Cotton (*Gossypium hirsutum*, variety: Zhongmian 36) was grown under controlled greenhouse conditions (25/18 °C day/night temperature, 16L/8D photoperiod, 50–60% relative humidity, light intensity of 300 μmol·m^−2^·s^−1^, and ambient CO_2_ levels of 400 ppm). After a seven-day acclimatization period in a climate chamber (25 °C, 16L/8D photoperiod, 60% relative humidity, light intensity of 250 μmol·m^−2^·s^−1^, and CO_2_ levels maintained at 400 ppm), experiments involving the plants were conducted. *Tetranychus turkestani* mites were collected in June 2023 from the experimental fields of the College of Agriculture, Shihezi University. The mites were reared on cotton plants in a light incubator (25 °C, 16L/8D photoperiod, 60% relative humidity, light intensity of 200 μmol·m^−2^·s^−1^, and ambient CO_2_ levels) without exposure to any pesticides. To minimize potential environmental contaminants, the incubator was regularly disinfected with 75% ethanol, and fine mesh sieves were used to prevent the entry of natural predators. The rearing environment was routinely monitored to ensure cleanliness and environmental control.

### 2.2. Detection of Wolbachia Infection in T. turkestani

The total DNA of *T. turkestani* was extracted following the method described by Yang Kang et al. [18]. Specific primers targeting the *wsp* gene of *Wolbachia* were designed (see Table S1 in Supplementary Materials). The PCR reaction volume was 25 μL, containing 2.0 μL of DNA template, 14.8 μL of ddH_2_O, 2.5 μL of 10× buffer, 2.0 μL of dNTPs, 1.5 μL of MgCl_2_, 0.2 μL of Taq polymerase, and 0.5 μL each of forward and reverse primers (20 mmol/L). The PCR conditions were as follows: pre-denaturation at 94 °C for 2 min, 35 cycles of denaturation at 94 °C for 30 s, annealing at 55 °C for 45 s, and extension at 72 °C for 45 s, followed by a final extension at 72 °C for 5 min. PCR products (15–20 μL) were analyzed using 1% agarose gel electrophoresis.

### 2.3. Screening of Mite Strains with Different Wolbachia Infection Statuses

Cultivation of 100% *Wolbachia*-infected strains: Parthenogenetic backcrossing was employed to cultivate strains fully infected with *Wolbachia*. Fresh, intact kidney bean leaves were placed in Petri dishes (9 cm diameter) containing sponges. The leaves were divided into 3–5 compartments of approximately equal area using moist, absorbent cotton strips. A single unmated female deutonymph from the laboratory strain was placed in each compartment for parthenogenetic reproduction to produce male offsprings. Once the eggs developed into adult males, they were backcrossed with their mother. After two days of backcrossing, the mother was transferred to a new compartment to lay eggs. Seven days later, the mother was tested for *Wolbachia* infection via PCR. The offsprings of *Wolbachia*-infected females were subjected to the same process for four to five generations. Subsequently, three replicates were conducted for each treatment, with 50 test insects per replicate subjected to a PCR assay, and strains showing 100% *Wolbachia* infection were used for experiments [19] (Supplementary Figure S1).

Cultivation of *Wolbachia*-uninfected strains: Bean leaves were soaked in a 0.1% tetracycline solution for 24 h and then used to feed newly hatched infected larval mites. Three replicates were conducted for each treatment, with 50 test insects per replicate subjected to a PCR assay. Strains that showed a complete absence of infection were classified as *Wolbachia*-uninfected. Prior to the experiment, these uninfected strains were reared for over 10 generations without exposure to tetracycline to eliminate any potential adverse effects of the antibiotic (Supplementary Figure S2) [20].

### 2.4. Infection Process of Mite Strains with Different Wolbachia Infection Statuses on Host Plants

The selected *T. turkestani* strains were acclimated to cotton plants. Five 21-day-old cotton plants were selected for each strain, with three leaves per plant. Mites aged 3 ± 1 days were placed on the experimental cotton leaves, with 30 mites per leaf for a 7-day infection period. Moist, absorbent cotton was used to block mite escape from the petioles. The number of adult mites was checked daily, and any escaped or dead mites were promptly replaced. Each treatment was performed in triplicate. After 7 days of infection, cotton leaves were collected. Prior to sampling, mites were gently removed from the leaf surfaces using fine brushes, ensuring that no residual mite mouthparts or other body parts remained on the leaves. The leaves were quickly excised (within 5 s) using sterilized scissors, immediately flash frozen in liquid nitrogen for 10 min, and then transferred to a −80 °C ultra-low temperature freezer for storage, which were then used for metabolomics sequencing analysis.

### 2.5. Metabolomics Sequencing of Host Plants

Cotton leaves infected by *Wolbachia*-infected mites were labeled as YJ, and those infected by *Wolbachia*-uninfected mites were labeled as WJ. After gradual thawing at 4 °C, appropriate amounts of samples were mixed with a pre-cooled methanol/acetonitrile/water solution (2:2:1, *v*/*v*), vortexed, ultrasonicated at a low temperature for 30 min, and left to stand at −20 °C for 10 min. The samples were centrifuged at 14,000× *g* at 4 °C for 20 min. The supernatant was collected and vacuum-dried. For mass spectrometry analysis, the dried samples were reconstituted with 100 μL of an acetonitrile–water solution (1:1, *v*/*v*), vortexed, and centrifuged at 14,000× *g* at 4 °C for 15 min, and the supernatant was used for analysis. Samples were separated using an Agilent 1290 Infinity LC ultra-high-performance liquid chromatography (UHPLC) system equipped with the HILIC column. The primary and secondary spectra were collected using an AB Triple TOF 6600 mass spectrometer (AB SCIEX, Framingham, MA, USA). Mass spectrometry analysis was performed in both positive and negative electrospray ionization (ESI) modes. The raw data were converted to the mzXML format using ProteoWizard (V3.0.7414), and the XCMS software (V3.18.0) was used for peak alignment, retention time correction, and peak area extraction. The extracted data were subjected to metabolite structure identification, data preprocessing, and experimental data quality evaluation, followed by data analysis. Metabolites with a variable importance in projection (VIP) > 1 and a *p* value < 0.05 were considered as differential metabolites.

### 2.6. Transcriptomic Sequencing of T. turkestani

Sample collection and numbering: The uninfected strains of *T. turkestani* (free of *Wolbachia* infection) were selected. The sample of eggs (E), larvae (L), nymphs (N), adult females (A_F), and adult males (A_M) were collected into 1.5 mL sterile centrifuge tubes, with approximately 50 mg per tube, in three replicates. For the *Wolbachia*-infected strains, the samples of eggs (E_W), larvae (L_W), nymphs (N_W), adult females (A_W_F), and adult males (A_W_M) were similarly collected. The collected samples were immediately placed in liquid nitrogen and then rapidly transferred to a −80 °C ultra-low temperature freezer for storage.

Total RNA was extracted following the protocols provided by Invitrogen Trizol Reagent. Libraries were prepared using 1 µg of total RNA. Poly(A) mRNA was isolated using Oligo(dT) beads and fragmented using divalent cations. cDNA synthesis was performed using the NEBNext UltraTM RNA Library Prep Kit (NEB, Ipswich, MA, USA). The purified double-stranded cDNA was end-repaired, dA tailed, and ligated with adapters. Size selection of adaptor-ligated DNA was performed using DNA Clean Beads. PCR amplification of each sample was carried out using P5 and P7 primers, and PCR products were verified. The libraries, indexed with different barcodes, were multiplexed and loaded onto the Illumina HiSeq 3000 platform (Illumina, San Diego, CA, USA) for sequencing, following the manufacturer’s instructions, using a 2 × 150 paired-end (PE) configuration. Quality control (QC) was performed using fastp. High-quality data were subjected to downstream analysis. QC statistics, including total reads, raw data, raw depth, error rates, and Q30 percentages, were calculated. Clean reads were aligned to the *T. urticae* reference genome using the Hisat2 (v2.2.1) software. Gene and isoform counts were estimated using HTSeq (v0.6.1), and FPKM (fragments per kilobase of transcript per million mapped reads) values were calculated based on gene length. Differential gene expression analysis was conducted using DESeq2 (V1.26.0) with the following criteria: |log2FoldChange| > 1 and *p*-value < 0.05.

### 2.7. Influence of Wolbachia on the Lifespan and Developmental Duration of Mites

Influence of *Wolbachia* on the lifespan of mites: Due to the susceptibility of male mites to drowning during rearing, only the lifespan of female mites was observed. A total of 90 quiescent stage III female mites were selected. After 12 h, newly hatched females were removed, and females hatched within the next 8 h were transferred to fresh leaves. Four females of the same infection status were placed on each leaf, with 13 leaves per infection status, totaling 52 females per strain. To ensure adequate nutrition, mites were transferred to fresh leaves every three days. The number of dead females was recorded daily, and deceased females were removed until all mites had died. Survival rates at different stages were compared using the Kaplan–Meier method and the log-rank test in SPSS 20.0. The hazard ratio (HR) was calculated using the log-rank test. All statistical tests were adjusted for multiple comparisons. Statistical analyses were performed using SPSS 20.0, and figures were generated using GraphPad Prism 9.0.

Influence of *Wolbachia* on the developmental duration of mites: Infected and uninfected female mites were placed on fresh leaves to oviposit for 8 h. After eight hours, the eggs were transferred to fresh leaves partitioned with absorbent paper, with one egg per section. Development stages were observed every 12 h, and the duration of each instar was recorded. Each treatment was conducted in three replicates, with 30 samples per replicate. For developmental ephemeral data and transformed data that did not follow a normal distribution, as determined by the Shapiro-Wilk test, non-parametric Mann–Whitney U tests were conducted for statistical analysis using SPSS 20.0.

### 2.8. Comparison of Leaf Damage Area

After seven days of infection, all mites and eggs were removed from the cotton leaves. The leaves were flattened using a thin glass plate, and images were captured with a Nikon camera to assess the feeding damage caused by spider mites. Each treatment was conducted in three replicates, with four test leaves per replicate. The damaged leaf area (mm^2^) was estimated using the “Compu Eye, Leaf & Symptom Area” software (http://www.ehabsoft.com/CompuEye/LeafSArea) (accessed on 21 September 2024 ) [21].

## 3. Results

### 3.1. Phenotypic Analysis of Cotton Leaves Infected by Different Mites

**Figure 1:**
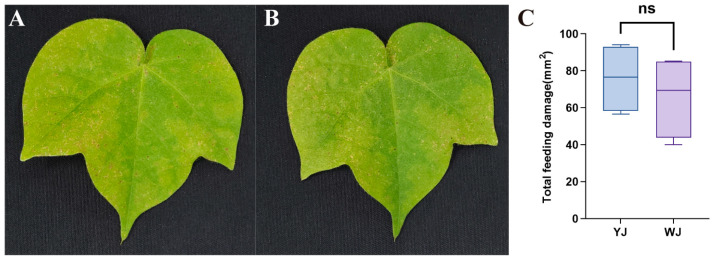
Phenotype of cotton leaves infected by mites with different *Wolbachia* infection statuses: (**A**) cotton leaves after YJ infection, (**B**) cotton leaves after WJ infection, and (**C**) comparison of the damaged leaf area. ns: not significant.

The impact of *Wolbachia* within mites on the phenotypic damage of cotton leaves is shown in Figure 1. There were significant differences in the extent and type of damage caused by mites infected and uninfected with *Wolbachia* after feeding on cotton. Mites infected with *Wolbachia* caused rust-colored necrotic spots on the leaf surface, resembling symptoms of plant pathogens (Figure 1A), whereas mites without *Wolbachia* infection caused chlorotic spots on the leaf surface (Figure 1B). The leaf damage area in the YJ group was 75.93 ± 9.33 mm^2^, compared to 66.04 ± 11.16 mm^2^ in the WJ group. An independent sample *t*-test revealed no statistically significant difference between the two groups (t (6) = 0.680, *p* = 0.52). However, there was no significant differences in the damaged leaf area between the *Wolbachia*-infected and *Wolbachia*-uninfected mite strains (Figure 1C).

### 3.2. Metabolomic Analysis of Cotton Leaves Infected by Different Mites

**Figure 2:**
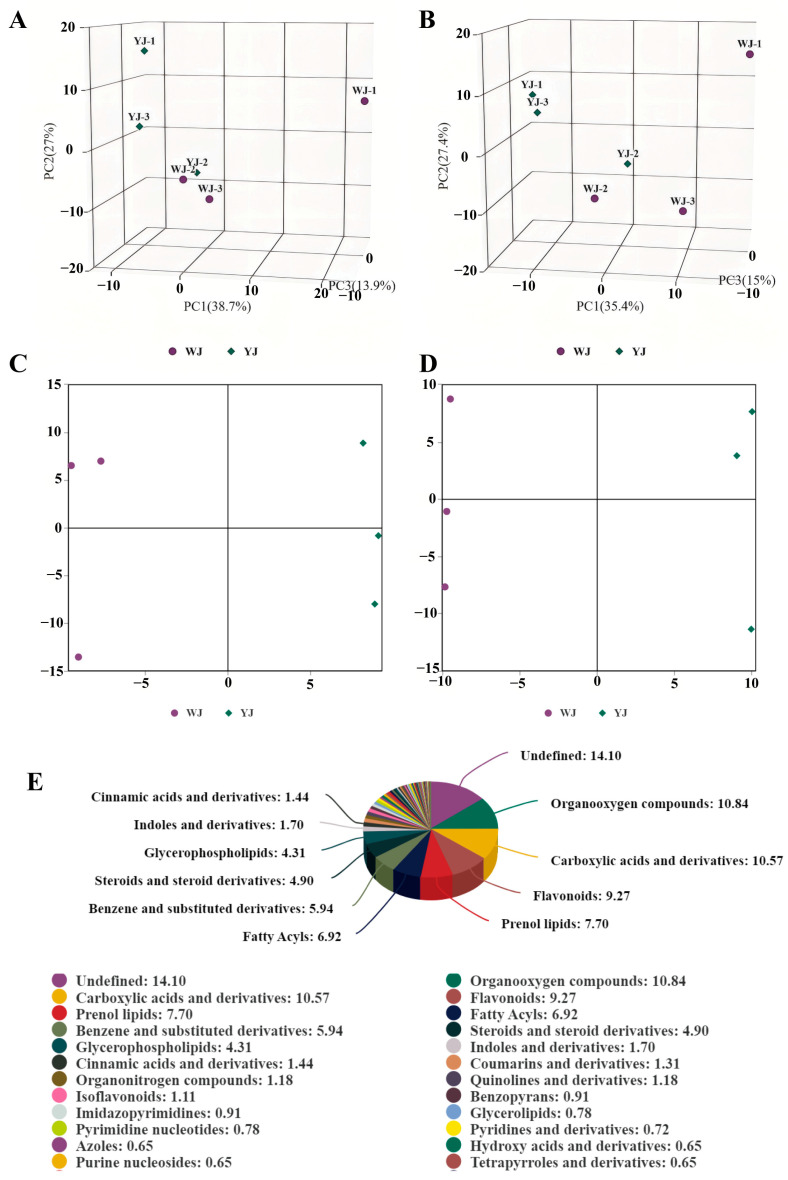
PCA of YJ and WJ metabolomic profiles: (**A**) PCA of variance-stabilized counts in the positive ion mode for YJ vs. WJ, (**B**) PCA of variance-stabilized counts in the negative ion mode for YJ vs. WJ, (**C**) OPLS-DA of the positive ion mode in YJ vs. WJ, (**D**) OPLS-DA of the negative ion mode in YJ vs. WJ, and (**E**) classification of differential metabolites.

Non-targeted metabolomic sequencing was performed on cotton leaves infected by *Wolbachia*-infected (YJ) and *Wolbachia*-uninfected (WJ) mites. To evaluate the metabolic differences between YJ and WJ and the variability within each sample, principal component analysis (PCA) was conducted on the metabolites in both positive and negative ion modes. The analysis showed that the contribution rates of PCA1 and PCA2 were 57.6% in the positive ion mode and 60.4% in the negative ion mode (Figure 2A,B). In both modes, the samples clustered within the 95% confidence interval, and samples from different groups were distinctly separated, indicating significant metabolic differences between the groups. The QC overlap indicated high similarity and good quality among samples. Orthogonal partial least squares discriminant analysis (OPLS-DA) was performed on the comparative groups. The OPLS-DA score plots showed that the R2 values in both positive and negative ion modes were greater than 0.71, and the Q2 values were greater than 0.67, indicating good predictive ability of the model. Samples were clustered within the 95% confidence interval, with clear separation between groups (Figure 2C,D). LC-MS/MS sequencing identified a total of 1532 differential metabolites, with 883 metabolites identified in the positive ion mode and 649 metabolites in the negative ion mode (Figure 2E).

**Figure 3:**
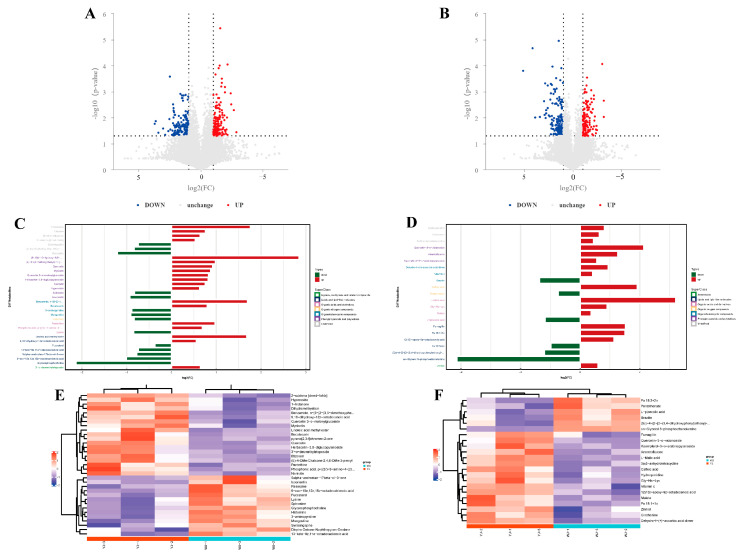
Differential metabolite analysis: (**A**) differential metabolite volcano plot of YJ vs. WJ in the positive ion mode, (**B**) differential metabolite volcano plot of YJ vs. WJ in the negative ion mode, (**C**) fold changes in significant differential metabolites in the positive ion mode of YJ vs. WJ, (**D**) fold changes in significant differential metabolites in the negative ion mode of YJ vs. WJ, (**E**) hierarchical clustering heatmap in the positive ion mode of YJ vs. WJ, and (**F**) hierarchical clustering heatmap in the negative ion mode of YJ vs. WJ.

Significant differential metabolites were identified based on the variable importance in projection (VIP) values from the OPLS-DA model and Student’s *t*-test *p*-values. Metabolites with VIP > 1 and *p* < 0.05 were considered significantly different. A total of 55 differential metabolites were identified using fold change (FC) thresholds of >1.5 or <0.67 (Figure 3A,B), including 33 in the positive ion mode and 22 in the negative ion mode (Figure 3C,D) (see Table S2 in Supplementary Materials). Differential compounds were classified, revealing that the metabolites differing between the YJ and WJ samples were primarily lipids (thirteen types); phenylpropanoids and polyketides (thirteen types); organic heterocyclic compounds (seven types); organic acids and derivatives (seven types); organic oxygen compounds (two types); organic nitrogen compounds (one type); lignans, neolignans and related compounds (one type); and benzene derivatives (one type). The hierarchical clustering analysis of YJ vs. WJ samples showed that, in the positive ion mode, 19 metabolites were upregulated, and 14 were downregulated in the YJ group. In the negative ion mode, sixteen metabolites were upregulated, and six were downregulated in the YJ group (Figure 3E,F).

In cotton leaves infected with *Wolbachia*, lysine was downregulated, which might have led to a deficiency in the essential amino acid lysine required by the cotton spider mite, negatively affecting its lifespan and fitness. L-malic acid, known to reduce fat content in insects, was upregulated with a fold change of 9.25 in *Wolbachia*-infected cotton leaves, potentially decreasing the essential fat content required by the spider mite, impairing its fat storage processes, and compromising its survival. Glycerophosphocholine (GPC) was significantly downregulated with a fold change of 0.23, and sn-glycerol-3-phosphoethanolamine (PE) was significantly downregulated with a fold change of 0.06. These metabolites are associated with the formation of cellular membranes, and their downregulation suggested that *Wolbachia* might have compromised the integrity and function of the plant cell membrane structures, thereby assisting the host spider mite in better accessing the intracellular sap of plant cells. Among secondary metabolites, flavonoids such as quercetin and myricetin were significantly upregulated with fold changes of 4.4 and 1.83, respectively. The upregulation of these secondary metabolites might have enhanced the defensive capability of cotton leaves against spider mite infection.

**Figure 4:**
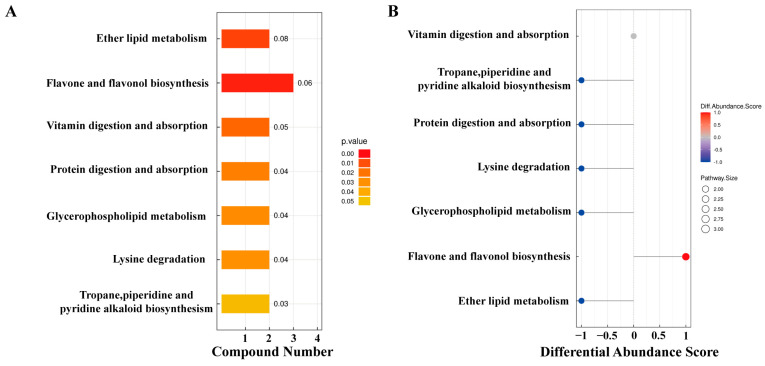
KEGG enrichment analysis of differential metabolites: (**A**) pathway enrichment map of differential metabolites in YJ vs. WJ and (**B**) abundance difference score plot for differential metabolite enrichment pathways in YJ vs. WJ.

The KEGG pathway analysis of differential metabolites identified 15 metabolites enriched in seven pathways (Figure 4). Among these, ether lipid metabolism and flavonoid and flavonol biosynthesis pathways exhibited extremely significant differences in YJ vs. WJ comparison (*p* < 0.01). The protein digestion and absorption pathway enriched two metabolites: histamine and lysine. The flavonoid and flavonol biosynthesis pathway enriched three metabolites: quercetin, myricetin, and quercetin 3-O-malonylglucoside. The vitamin digestion and absorption pathway enriched two metabolites: pantothenic acid and vitamin C. The tropane, piperidine, and pyridine alkaloid biosynthesis pathway enriched lysine and pipecolic acid. The lysine degradation pathway enriched lysine and pipecolic acid as well. Both glycerophospholipid metabolism and ether lipid metabolism pathways enriched (GPC) and (PE).

**Figure 5:**
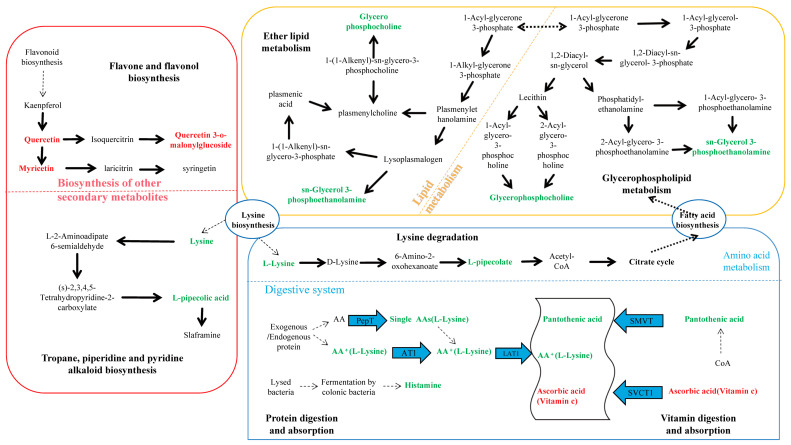
KEGG enrichment pathway analysis for differential metabolites. This figure illustrates lipid metabolism, secondary metabolism, and protein and amino acid metabolism within cotton cells. Lysine biosynthesis connected lysine degradation to alkaloid synthesis, with lysine degradation entering the citric acid cycle via the pipecolic acid pathway. Glycerophospholipid metabolism interacted with ether lipid metabolism through 1-acylglycerol-3-phosphate, and both glycerophosphocholine (GPC) and sn-glycerol-3-phosphoethanolamine (PE) were significantly downregulated in both glycerophospholipid metabolism and ether lipid metabolism pathways. Flavonoids such as quercetin and myricetin were significantly upregulated among secondary metabolites, while L-lysine and pantothenic acid were significantly downregulated in the protein digestion and absorption pathway.

Among the 15 differential metabolites, lysine was annotated in three pathways: lysine degradation; protein digestion and absorption; and tropane, piperidine, and pyridine alkaloid biosynthesis. GPC and PE were annotated in both glycerophospholipid metabolism and ether lipid metabolism pathways. Figure 5 summarized the metabolic cycling pathways of cotton leaves infected by *Wolbachia*-infected spider mites, including lipid metabolism, secondary metabolism, and protein and amino acid metabolism. Lysine biosynthesis linked lysine degradation to alkaloid biosynthesis, with lysine degradation entering the citric acid cycle via the pipecolic acid pathway. Glycerophospholipid metabolism interacted with ether lipid metabolism through 1-acylglycerol-3-phosphate, and both GPC and PE were significantly downregulated in these two pathways. Among secondary metabolites, flavonoids such as quercetin and myricetin were significantly upregulated. In the protein digestion and absorption pathway, metabolites like L-lysine and pantothenic acid were significantly downregulated.

### 3.3. The Influence of Wolbachia on the Lifespan and Developmental Duration of the Host

**Figure 6:**
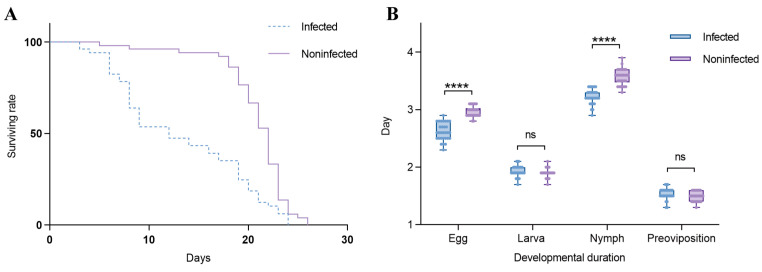
Effects of *Wolbachia* on the reproductive capacity and fitness of spider mites: (**A**) survival rate and (**B**) duration of development. ns: not significant. ****: *p* < 0.001.

Effects of *Wolbachia* on the lifespan of *T. turkestani*: Female mites infected with *Wolbachia* began to die on the 3rd day after oviposition, while uninfected females started to die on the 5th day. Subsequently, the mortality rate of *Wolbachia*-infected female mites accelerated, with mass mortality occurring from the 6th day onwards. In contrast, uninfected female mites exhibited mass mortality starting on the 18th day. The lifespan difference between the two groups was extremely significant (χ^2^ = 25.53, df = 1, *p* < 0.001). The hazard ratio (HR) for the infected group compared to the uninfected group was 2.347, with a 95% confidence interval (CI) ranging from 1.524 to 3.613 (Figure 6A).

In this study, the Shapiro–Wilk test was used to assess the normality of the data for the infected and uninfected groups at each developmental stage. For the egg stage, the W statistics were 0.9398 (*p* = 0.0896) for the infected group and 0.8621 (*p* = 0.0011) for the uninfected group. In the larval stage, the W statistics were 0.9039 (*p* = 0.0105) for the infected group and 0.8482 (*p* = 0.0006) for the uninfected group. For the nymph stage, the W statistics were 0.9159 (*p* = 0.0211) for the infected group and 0.9457 (*p* = 0.1297) for the uninfected group. During the pre-oviposition period, the W statistics were 0.8800 (*p* = 0.0028) for the infected group and 0.8578 (*p* = 0.0009) for the uninfected group. These results indicate that at least one group in each developmental stage did not meet the assumption of normality (sample size per group: n = 30). Influence of *Wolbachia* on the developmental duration of *T. turkestani*: There were no significant differences in the larval stage (1.93 ± 0.10 days for infected females vs. 1.90 ± 0.08 days for uninfected females; *p* = 0.189) and the pre-oviposition period (1.54 ± 0.10 days for infected females vs. 1.49 ± 0.08 days for uninfected females; *p* = 0.056) between *Wolbachia*-infected and *Wolbachia*-uninfected female mites. However, the egg stage (2.62 ± 0.15 days for infected females vs. 2.97 ± 0.11 days for uninfected females) and the nymphal stage (3.23 ± 0.13 days for infected females vs. 3.57 ± 0.14 days for uninfected females) were significantly shorter in infected mites (*p* < 0.01). The overall developmental duration from the egg stage to the pre-oviposition period was also significantly different between infected (9.31 ± 0.25 days) and uninfected (9.93 ± 0.19 days) female mites (*p* < 0.01) (Figure 6B). The experimental results indicated that *Wolbachia* infection shortened both the lifespan and developmental duration of *T. turkestani*.

### 3.4. Wolbachia Enhanced the Expression of Detoxification Metabolism Genes T. turkestani

**Figure 7:**
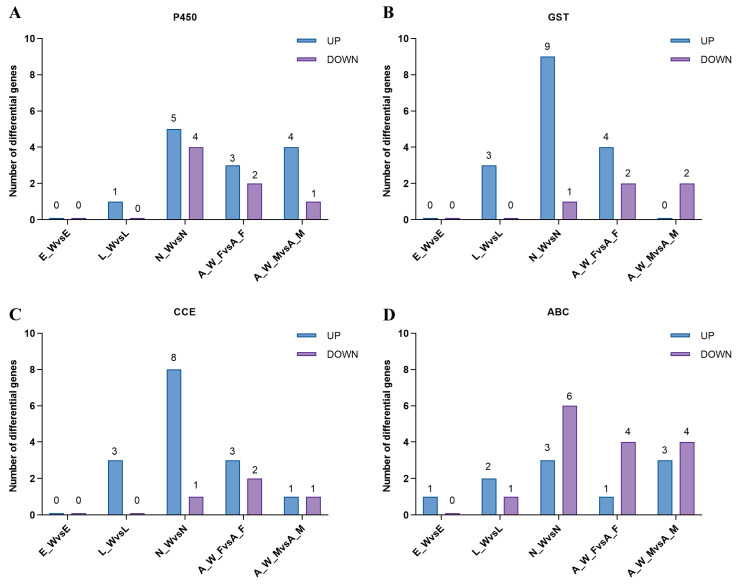
Differential expression of detoxification metabolism genes in different developmental stages of spider mite. E_W vs. E: egg stage; L_W vs. L: larval stage; N_W vs. N: nymphal stage; A_W_F vs. A_F: adult female; A_W_M vs. A_M: adult male: (**A**) cytochrome P450, (**B**) glutathione S-transferase, (**C**) carboxylesterase, and (**D**) ABC transporter.

Following *Wolbachia* infection, the differential expression of genes involved in detoxification metabolism was observed across various developmental stages. This included major detoxification gene families such as cytochrome P450, glutathione S-transferase, carboxylesterase, and ABC transporters (Figure 7). During the egg stage (E_W vs. E), one gene was upregulated, and none were downregulated; during the larval stage (L_W vs. L), nine genes were upregulated, and one gene was downregulated. During the nymph stage (N_W vs. N), 25 genes were upregulated, and 12 genes were downregulated; in adult females (A_W_F vs. A_F), 11 genes were upregulated, and 10 genes were downregulated. In adult males, eight genes were upregulated, and eight genes were downregulated (see Supplementary Table S3). The number of upregulated detoxification metabolism genes exceeded the number of downregulated genes across different developmental stages, indicating that *Wolbachia* infection enhanced the detoxification capacity of the host mites.

## 4. Discussion

This study utilized metabolomics to investigate the physiological and metabolic changes in cotton leaves following infestation by spider mites both infected and uninfected with *Wolbachia*. The analysis identified key differential metabolites, including glycerophosphocholine, glycerophosphoethanolamine, malic acid, quercetin, amino acids, lignans, etc. KEGG pathway analysis revealed significant differences in pathways related to flavonoid and flavonol biosynthesis, glycerophospholipid metabolism, and the biosynthesis of tropane, piperidine, and pyridine alkaloids. These metabolic alterations suggested that *Wolbachia* infection activates cotton defense mechanisms, subsequently impacting the growth and development of spider mites.

*Wolbachia* infection induced the production of secondary metabolites in cotton, particularly flavonoids and alkaloids, which play crucial roles in plant defense. Flavonoids such as quercetin, kaempferol, amentoflavone, and myricetin function as phytoalexins and antioxidants, mitigating biotic and abiotic stresses by scavenging reactive oxygen species (ROS). Notably, the levels of myricetin and quercetin increased by 1.83-fold and 4.4-fold, respectively, indicating their direct role in enhancing cotton’s defense system against spider mite infestation. These findings aligned with previous studies, highlighting the protective role of flavonoids in plant–insect interactions [22,23,24,25,26]. However, the direct causal relationship between *Wolbachia* infection and the upregulation of these metabolites remained to be experimentally validated, for example, through targeted gene knockout or metabolite supplementation experiments.

In addition to influencing secondary metabolism, *Wolbachia* infection disrupted lipid metabolism in cotton. Key lipid metabolites, including sn-glycerol-3-phosphoethanolamine (PE) and glycerophosphocholine (GPC), are essential for maintaining the integrity and function of plant cell membranes [27,28]. The downregulation of PE and GPC suggested that *Wolbachia* infection may compromise the integrity of plant cell membranes, facilitating spider mites’ access to intracellular fluids and enhancing their nutrient acquisition. This may represent a strategic adaptation by *Wolbachia*.

*Wolbachia* infection significantly influenced the physiology of spider mites, particularly affecting their growth, reproduction, and detoxification abilities. In cotton leaves, the downregulation of lysine and histamine likely led to lysine deficiency in spider mites that fed on these leaves, consequently shortening their lifespan [29]. Moreover, the marked increase in L-malate, a key organic acid involved in energy metabolism, may interfere with fat storage processes in spider mites, further compromising their survival [30,31,32]. These metabolic alterations suggested that *Wolbachia* infection imposes physiological costs on spider mites, potentially counter-balancing the advantages gained from enhanced detoxification capabilities.

Indeed, *Wolbachia* infection up-regulated several detoxification-related genes in spider mites, particularly within the three major detoxification gene families, thereby enhancing their resistance to plant secondary metabolites [33]. Although this increased detoxification capacity improves spider mites’ adaptability to host plants, it also incurs an energy cost, necessitating trade-offs between growth, reproduction, and survival [34,35]. The observed reduction in spider mite lifespan in this study may reflect these trade-offs, highlighting the complex interactions among *Wolbachia*, spider mites, and cotton plants.

**Figure 8:**
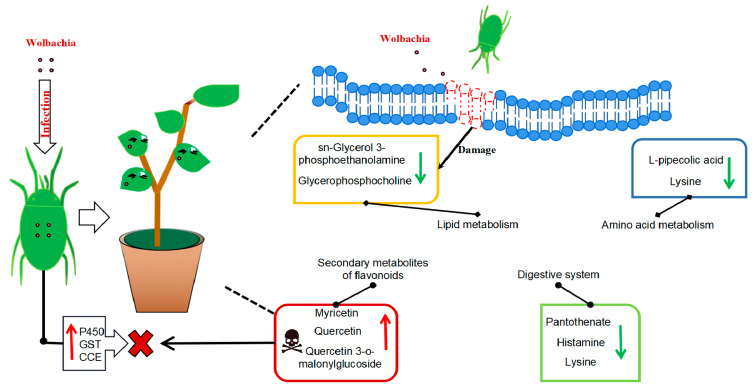
Metabolic profile of cotton leaves infected by *Wolbachia*-infected mites: *Wolbachia* infection that induced secondary metabolites in cotton led to the activation of flavonoid and flavonol biosynthesis and tropane, piperidine, and pyridine alkaloid biosynthesis, resulting in a significant upregulation of flavonoid compounds such as quercetin, enhancing the plant defense. It impairs lipid metabolism, resulting in a significant downregulation of lipid metabolites, such as sn-glycerol-3-phosphoethanolamine and glycerophosphocholine, and disrupting the integrity and functionality of plant cell membranes, potentially facilitating mite feeding. It also disrupts the growth and reproduction of mites via the down-regulation of lysine, leading to a deficiency in essential amino acid supply and a significant increase in L-malic acid, which damaged the fat storage process in mites and shortened their lifespan. *Wolbachia* infection induced the upregulation of numerous detoxification metabolic genes in mites, enhancing their ability to detoxify plant secondary metabolites.

In conclusion, this study demonstrated that *Wolbachia* infection induces multifaceted metabolic changes in cotton, influencing both plant defense mechanisms and spider mite physiology. While the up-regulation of secondary metabolites and the disruption of lipid metabolism may enhance plant resistance, the physiological costs to spider mites, including reduced lifespan and impaired growth, indicate a complex role for *Wolbachia* in plant–insect interactions (Figure 8). However, the causal relationships between *Wolbachia* infection, metabolic changes, and the observed phenotypic effects are not yet fully understood. Future research employing targeted gene manipulation, multi-omics approaches, and larger sample sizes will be essential to unravel these complex interactions. This study provides valuable insights into the molecular mechanisms underlying endosymbiont-mediated plant defense and insect–plant co-evolution, thereby contributing to the development of sustainable pest management strategies.

## Data Availability

The original contributions presented in this study are included in the article/Supplementary Material. Further inquiries can be directed to the corresponding authors.

## Supplementary Materials

The following supporting information can be downloaded at: https://www.mdpi.com/article/10.3390/microorganisms13030608/s1, Figure S1: Detection of *Wolbachia* infection status. Figure S2: Detection of *Wolbachia* uninfected status. Table S1: Primer sequences of the *Wolbachia WSP* gene. Table S2: Differentially expressed metabolites in cotton leaves infested by different mites. Table S3: Differential expression of detoxification metabolism genes in different developmental stages of spider mite.
