# Supplementary material for: Metabolomics Provides New Insights into the Mechanisms of Wolbachia-Induced Plant Defense in Cotton Mites
Source: Microorganisms. 2025 Mar 6;13(3):608. doi: 10.3390/microorganisms13030608 (PMC11944673; doi:10.3390/microorganisms13030608)
Supplement: Supplementary file 1 [file microorganisms-13-00608-s001.zip › microorganisms-3494558-supplementary.pdf]

Table S1. Primer sequences of the *Wolbachia WSP* gene

Primer name	Sequences	Size of the sequence band
WSP/F236	GACAGTTTAACAGCATTTTCAGGA	211 bp
WSP/R44	GTTTGATTTCTGGAGTTACATCAT	

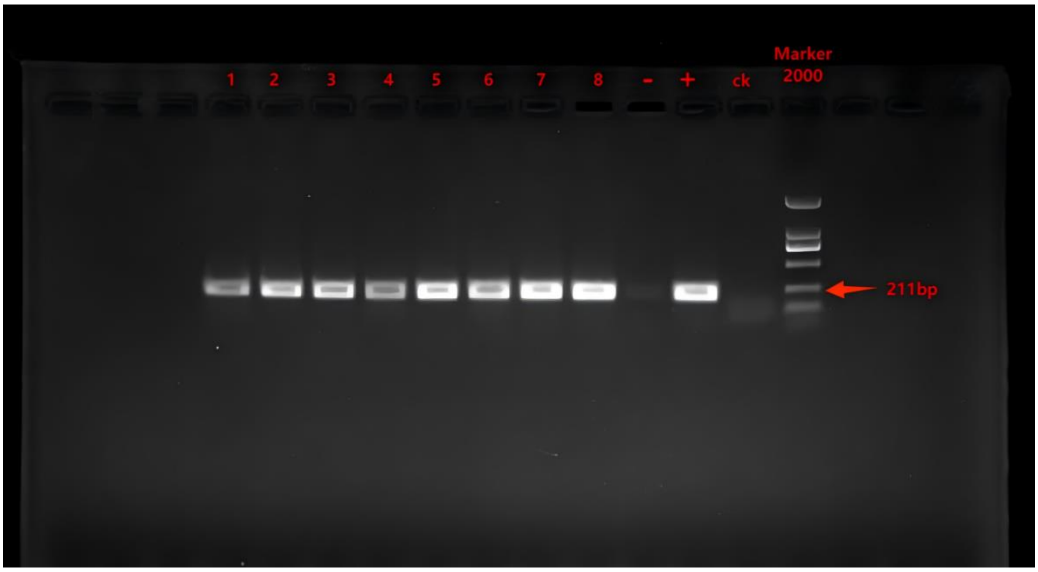

Figure S1. Detection of *Wolbachia* infection status

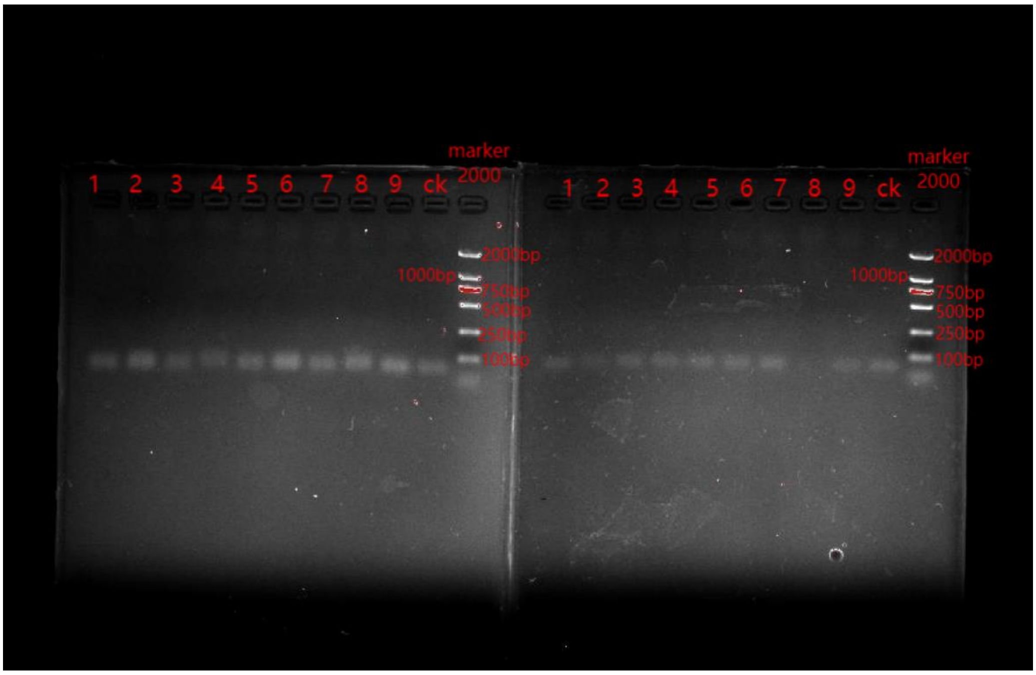

Figure S2. Detection of *Wolbachia* uninfected status

Table S2. Differentially expressed metabolites in cotton leaves infested by different mites

mode	Differential metabolite	SuperClass	VIP	FC	P Value	log_2FC
Positive	(9r,10r)-10-hydroxy-8,8-dimethyl-9-[(2s,3r,4s,5s,6r)-3,4,5-trihydroxy-6-(hydroxymethyl)oxan-2-yl]oxy-9,10-dihydropyrano[2,3-f]chromen-2-one	Phenylpropanoids and polyketides	1.53	7.21	3.70E-02	2.85
	1-Indanone		1.90	3.40	2.76E-02	1.76
	Benzamide, n-[5-[2-(3,5-dimethoxyphenyl)ethyl]-1h-pyrazol-3-yl]-4-[(3r,5s)-3,5-dimethyl-1-piperazinyl]-, rel-	Organoheterocyclic compounds	3.42		5.30E-03	1.70
	Linoleic acid methyl ester	Lipids and lipid-like molecules	3.24	3.24	2.86E-02	1.68
	(e)-3-(4-methoxyphenyl)-1-[2,4,6-trimethoxy-3-(3-methylbut-2-enyl)phenyl]prop-2-en-1-one	Phenylpropanoids and polyketides	1.04	1.97	2.77E-02	0.98
	Pantethine	Organic acids and derivatives	2.12	1.96	3.96E-02	0.97
	Quercetin	Phenylpropanoids and polyketides	5.25	1.89	8.23E-03	0.92
	Myricetin	Phenylpropanoids and polyketides	1.01	1.83	3.97E-02	0.88
	Quercetin 3-o-malonylglucoside	Phenylpropanoids and polyketides	1.10	1.77	1.66E-02	0.83
	Herbacetin-3,8-diglucopyranoside	Phenylpropanoids and polyketides	3.29	1.77	4.48E-02	0.82
	Becatecarin	Organoheterocyclic compounds	2.98	1.74	4.62E-02	0.80
	Etizolam		3.09	1.69	2.51E-02	0.76
	Narirutin	Phenylpropanoids and polyketides	3.84	1.69	1.52E-02	0.75
	Phosphonic acid, p-[(3r)-3-amino-4-[(3-hexylphenyl)amino]-4-oxobutyl]-	Organic acids and derivatives	1.01	1.62	4.58E-02	0.69
	3'-o-desmethyletoposide	Lignans, neolignans and related compounds	1.95	1.57	4.22E-02	0.65
	Dihydromethysticin		1.46	1.56	3.75E-02	0.64
	Hyperoside	Phenylpropanoids and polyketides	2.79	1.55	3.63E-02	0.63

	9,10-dihydroxy-12z-octadecenoic acid	Lipids and lipid-like molecules	1.02	1.47	2.17E-02	0.56
	2-sdahma [dmed-fahfa]		5.47	1.44	1.99E-03	0.53
	Fucosterol	Lipids and lipid-like molecules	1.08	0.77	1.03E-02	-0.37
	13-keto-9z,11e-octadecadienoic acid	Lipids and lipid-like molecules	6.17	0.60	3.67E-02	-0.74
	Syrosingopine		2.09	0.60	4.55E-02	-0.74
	5alpha-androstan-17beta-ol-3-one	Lipids and lipid-like molecules	1.53	0.58	4.97E-02	-0.78
	Spinosine	Phenylpropanoids and polyketides	1.56	0.56	2.62E-02	-0.83
	(2r,4'ar,5's,6'r,6'as,10'ar,10'br)-6'-(acetyloxy)-4'a,6'a,10'b-trimethyl-5''-oxo-decahydro-1'h-dispiro[oxirane-2,7'-naphtho[2,1-b]pyran-3',3''-oxolane]-5'-yl 2-methylpropanoate		1.11	0.56	2.91E-02	-0.83
	Histamine	Organic nitrogen compounds	6.85	0.56	2.86E-02	-0.83
	Lysine	Organic acids and derivatives	10.43	0.56	3.85E-02	-0.84
	3-aminopyridine	Organoheterocyclic compounds	6.56	0.54	3.49E-02	-0.89
	Mangostine	Organoheterocyclic compounds	1.29	0.53	2.22E-02	-0.91
	Isoorientin	Phenylpropanoids and polyketides	1.29	0.53	4.95E-02	-0.93
	9-oxo-10e,12z,15z-octadecatrienoic acid	Lipids and lipid-like molecules	3.95	0.50	1.65E-02	-1.01
	Reserpine		1.68	0.43	3.41E-02	-1.21
	Glycerophosphocholine	Lipids and lipid-like molecules	22.71	0.23	6.22E-03	-2.13
Negative	L-Malic acid	Organic acids and derivatives	6.99	9.25	2.22E-03	3.21
	Quercetin-3-o-vicianoside	Phenylpropanoids and polyketides	2.73	4.40	2.21E-02	2.14
	Caffeic acid	Organic oxygen compounds	1.67	3.78	2.34E-02	1.92

Fumagillin	Lipids and lipid-like molecules	1.45	2.87	4.83E-02	1.52
Fa 18:1+1o	Lipids and lipid-like molecules	4.44	2.85	1.54E-02	1.51
Amentoflavone	Phenylpropanoids and polyketides	1.16	2.40	1.67E-02	1.26
12(13)-epoxy-9z-octadecenoic acid	Lipids and lipid-like molecules	3.82	2.19	2.74E-03	1.13
Dehydro-l-(+)-ascorbic acid dimer	Organoheterocyclic compounds	4.73	1.92	2.89E-03	0.94
Gly-His-Lys	Organic acids and derivatives	3.59	1.88	3.36E-02	0.91
Hydroquinidine		3.65	1.76	4.08E-02	0.81
Cinchonine		2.30	1.56	3.62E-02	0.65
Zinniol	Benzenoids	3.43	1.52	4.32E-02	0.60
Kaempferol-3-o-arabinopyranoside	Phenylpropanoids and polyketides	1.12	1.48	4.50E-02	0.56
5a,6-anhydrotetracycline		4.12	1.36	5.26E-03	0.45
Vitamin c	Organoheterocyclic compounds	4.78	1.34	1.35E-02	0.42
Malate	Organic acids and derivatives	7.57	1.29	2.68E-02	0.37
Pantothenate	Organic oxygen compounds	1.54	0.60	4.01E-02	-0.74
Fa 18:3+2o	Lipids and lipid-like molecules	2.29	0.51	1.56E-02	-0.98
L-pipecolic acid	Organic acids and derivatives	2.99	0.44	2.47E-02	-1.18
(5z)-4-[2-[2-(3,4-dihydroxyphenyl)ethoxy]-2-oxoethyl]-5-ethylidene-6-[(2s,3r,4s,5s,6r)-3,4,5-trihydroxy-6-(hydroxymethyl)oxan-2-yl]oxy-4h-pyran-3-carboxylic acid	Lipids and lipid-like molecules	4.63	0.44	4.40E-02	-1.20
Brazilin	Organoheterocyclic compounds	1.02	0.39	4.72E-02	-1.37
sn-Glycerol 3-phosphoethanolamine	Lipids and lipid-like molecules	2.97	0.06	2.11E-05	-4.14

Table S3. Differential expression of detoxification metabolism genes in different developmental stages of spider mite

Comparative combination	category	gene_id	gene_description	log2FC	padj
E_WvsE	ABCs	novel.8707	PF00005:ABC transporter|PF01061:ABC-2 type transporter	1.54	2.98E-02
L_WvsL	P450s	107370433	cytochrome P450 2H2-like	2.23	4.45E-02
	GSTs	107369346	glutathione S-transferase 1-1-like	1.52	4.45E-03
		107369346	glutathione S-transferase 1-1-like	1.52	4.45E-03
		107370075	glutathione S-transferase 1%2C isoform C-like	1.31	3.11E-02
	CCEs	107369541	acetylcholinesterase-1-like	3.06	8.70E-03
		107365759	acetylcholinesterase-like	3.54	1.59E-02
		novel.10907	PF00135:Carboxylesterase family	2.51	4.55E-02
	ABCs	novel.3681	PF00005:ABC transporter|PF00664:ABC transporter transmembrane region	2.13	1.93E-02
		novel.10220	PF00005:ABC transporter|PF00664:ABC transporter transmembrane region	1.25	2.72E-02
		novel.5873	PF00005:ABC transporter|PF00664:ABC transporter transmembrane region	-7.82	1.61E-03
N_WvsN	P450s	107370433	cytochrome P450 2H2-like	2.59	2.53E-04
		novel.6027	PF00067:Cytochrome P450	2.37	6.07E-24
		107367653	cytochrome P450 2J6-like	1.92	1.36E-05
		107371733	cytochrome P450 1A1-like	1.07	1.86E-04
		107372119	cytochrome P450 2J6-like	1.01	4.91E-03
		107368628	cytochrome P450 18a1-like	-2.77	2.35E-03
		107362192	probable cytochrome P450 4ac1	-2.59	2.80E-03
		107361555	cytochrome P450 4V2-like	-2.28	2.79E-14
		107367093	cytochrome P450 2C15-like	-1.87	1.39E-02
	GSTs	107368271	glutathione S-transferase 1-1-like	2.28	2.20E-03
		107359332	glutathione S-transferase 1%2C isoform C-like	2.01	1.93E-02
		107368275	glutathione S-transferase 1%2C isoform C-like	1.96	2.23E-02
		107369346	glutathione S-transferase 1-1-like	1.84	1.89E-11
		107370075	glutathione S-transferase 1%2C isoform C-like	1.65	6.96E-06
		107360646	glutathione S-transferase Mu 3-like	1.60	3.01E-10
		novel.613	PF13417:Glutathione S-transferase, N-terminal domain	1.44	2.83E-05
		novel.5135	PF02798:Glutathione S-transferase, N-terminal domain|PF14497:Glutathione S-transferase, C-terminal domain	1.39	8.74E-03
		107361891	glutathione S-transferase 1%2C isoform C-like	1.37	5.85E-08
		novel.626	PF02798:Glutathione S-transferase, N-terminal domain|PF00043:Glutathione S-transferase, C-terminal domain	-5.91	1.99E-02
	CCEs	107359187	cholinesterase 2-like	4.16	1.42E-20

		107371827	cholinesterase-like	3.82	3.04E-34
		107369541	acetylcholinesterase-1-like	3.72	1.89E-11
		107367804	acetylcholinesterase-1-like	3.24	5.60E-08
		107365759	acetylcholinesterase-like	3.04	2.74E-04
		novel.10907	PF00135:Carboxylesterase family	2.87	9.90E-04
		novel.12826	PF00135:Carboxylesterase family	2.39	2.11E-02
		novel.13278	PF00135:Carboxylesterase family	1.92	2.10E-02
		107361446	acetylcholinesterase-like	-6.71	2.24E-03
	ABCs	novel.3681	PF00005:ABC transporter|PF00664:ABC transporter transmembrane region	2.28	3.20E-05
		novel.10220	PF00005:ABC transporter|PF00664:ABC transporter transmembrane region	1.56	3.37E-07
		107368416	ABC transporter G family member 20-like	1.41	3.84E-11
		novel.5873	PF00005:ABC transporter|PF00664:ABC transporter transmembrane region	-6.38	6.04E-04
		novel.11318	PF12698:ABC-2 family transporter protein|PF00005:ABC transporter	-5.33	1.29E-02
		107362311	ABC transporter B family member 7-like	-2.67	1.92E-36
		107364677	ABC transporter G family member 23-like	-1.69	1.39E-03
		107364633	ABC transporter G family member 23-like%2C transcript variant X4	-1.25	2.34E-03
		107359217	ABC transporter G family member 20-like	-1.24	5.12E-11
A_W_Fvs A_F	P450s	107359215	cytochrome P450 2J6-like	2.4	1.28E-19
		107360960	cytochrome P450 2J2-like	1.54	1.39E-03
		novel.6027	PF00067:Cytochrome P450	1.52	1.42E-11
		107370208	cytochrome P450 3A8-like	-2.89	2.20E-08
		107361047	cytochrome P450 2J6-like	-2.20	1.47E-02
	GSTs	107369780	glutathione-independent glyoxalase DJR-1.1-like	1.55	4.07E-02
		107361891	glutathione S-transferase 1%2C isoform C-like	1.51	2.06E-08
		107364624	glutathione S-transferase omega-1-like	1.45	1.25E-02
		107360789	glutathione S-transferase Mu 1-like	1.39	6.86E-03
		novel.613	PF13417:Glutathione S-transferase, N-terminal domain	1.34	3.71E-04
		novel.626	PF02798:Glutathione S-transferase, N-terminal domain|PF00043:Glutathione S-transferase, C-terminal domain	-5.46	1.36E-02
		107368272	glutathione S-transferase 1%2C isoform C-like	-5.45	1.13E-02
	CCEs	novel.12980	PF00135:Carboxylesterase family	3.69	5.00E-02
		107359187	cholinesterase 2-like	2.1	4.61E-08
		107361446	acetylcholinesterase-like	-6.72	7.68E-06
		107359355	putative inactive carboxylesterase 4	-3.04	4.80E-04
	ABCs	novel.13981	PF00005:ABC transporter|PF12698:ABC-2 family transporter protein	2.08	5.03E-04
		novel.5873	PF00005:ABC transporter|PF00664:ABC	-6.05	1.15E-09

			transporter transmembrane region		
		novel.11318	PF12698:ABC-2 family transporter protein|PF00005:ABC transporter	-4.95	3.83E-05
		107366618	ABC transporter G family member 23-like	-3.61	6.08E-04
		107362311	ABC transporter B family member 7-like	-1.26	2.05E-03
A_W_Mvs A_M	P450s	107360212	cytochrome P450 4C1-like	1.6	5.70E-03
		107365376	cytochrome P450 4C1-like	1.41	7.87E-06
		107368633	cytochrome P450 18a1-like	1.17	8.91E-03
		107360669	cytochrome P450 4c21-like%2C transcript variant X4	1.02	2.38E-02
		107364290	cytochrome P450 2C15-like	0.93	1.98E-05
		107370208	cytochrome P450 3A8-like	-1.03	4.04E-06
	GSTs	novel.626	PF02798:Glutathione S-transferase, N-terminal domain|PF00043:Glutathione S-transferase, C-terminal domain	-5.76	2.15E-03
		107360606	glutathione S-transferase Mu 1-like	-1.32	1.86E-03
	CCEs	107370066	carboxylesterase 4A-like	1.97	3.74E-04
		107361446	acetylcholinesterase-like	-7.74	8.66E-08
	ABCs	107360159	ABC transporter G family member 23-like	1.6	4.40E-02
		novel.8707	PF00005:ABC transporter|PF01061:ABC-2 type transporter	1.24	7.55E-05
		107369561	ABC transporter G family member 20-like	1.2	9.06E-06
		novel.5873	PF00005:ABC transporter|PF00664:ABC transporter transmembrane region	-6.70	4.48E-06
		novel.11318	PF12698:ABC-2 family transporter protein|PF00005:ABC transporter	-2.89	5.00E-03
		107366618	ABC transporter G family member 23-like	-2.02	9.00E-03
		novel.9181	PF00005:ABC transporter|PF00664:ABC transporter transmembrane region	-1.75	4.64E-02
